# Pattern of *RECK* CpG methylation as a potential marker for predicting breast cancer prognosis and drug-sensitivity

**DOI:** 10.18632/oncotarget.8620

**Published:** 2016-04-06

**Authors:** Gongping Shi, Yoko Yoshida, Kanako Yuki, Tomomi Nishimura, Yukiko Kawata, Masahiro Kawashima, Keiko Iwaisako, Kiyotsugu Yoshikawa, Junichi Kurebayashi, Masakazu Toi, Makoto Noda

**Affiliations:** ^1^ Department of Molecular Oncology, Kyoto University Graduate School of Medicine, Yoshida-Konoe-cho, Sakyo-ku, Kyoto 606-8501, Japan; ^2^ Laboratory for Malignancy Control Research, Medical Innovation Center, Kyoto University Graduate School of Medicine, Yoshida-Konoe-cho, Sakyo-ku, Kyoto 606-8501, Japan; ^3^ Department of Breast Surgery, Kyoto University Graduate School of Medicine, Yoshida-Konoe-cho, Sakyo-ku, Kyoto 606-8501, Japan; ^4^ Department of Target Therapy and Oncology, Kyoto University Graduate School of Medicine, Yoshida-Konoe-cho, Sakyo-ku, Kyoto 606-8501, Japan; ^5^ Department of Breast and Thyroid Surgery, Kawasaki Medical School, Kurashiki, 701-0192, Japan

**Keywords:** RECK, DNA methylation, CpG island, breast cancer, entinostat

## Abstract

The membrane-anchored glycoprotein RECK negatively regulates multiple metalloproteinases and is frequently downregulated in tumors. Forced RECK expression in cancer cells results in suppression of tumor angiogenesis, invasion, and metastasis in xenograft models. A previous methylome study on breast cancer tissues detected inverse correlation between *RECK* CpG methylation (in an intron-1 region) and relapse-free survival. In this study, we focused on another region of the *RECK* CpG island (a promoter/exon-1 region) and found an inverse correlation between its methylation and *RECK*-inducibility by an HDAC inhibitor, MS275, among a panel of breast cancer cell lines (n=15). In clinical samples (n=62), *RECK* intron-1 methylation was prevalent among luminal breast cancers as reported previously (26 of 38 cases; 68%) and particularly enriched in tumors of the ER+PR- subclass (10 of 10 cases) and of higher histological grades (Grade 2 and 3; 28 of 43 cases; *P*=0.006). In about a half of these cases, promoter/exon-1 methylation was absent, and hence, *RECK* may be inducible by certain drugs such as MS275. Our results indicate the value of combined use of two *RECK* methylation markers for predicting prognosis and drug-sensitivity of breast cancers.

## INTRODUCTION

Breast cancer, a major health concern worldwide [1], exhibits disparate clinical behaviors and patient outcomes [2]. Ductal carcinoma *in situ* (DCIS) is a non-invasive breast lesion that accounts for 10-25% of all breast neoplasms; DCIS is not life-threatening but a risk factor for, and potential precursor of, invasive cancers. The most common histological type of breast cancers, accounting for ~80% of the cases, is invasive ductal carcinoma (IDC). IDC has been subgrouped based on immunohistochemical detection of several markers such as estrogen receptor (ER), progesterone receptor (PR), and epidermal growth factor receptor 2 (HER2). A large fraction (70–75%) of IDCs are positive for ER and classified as luminal cancers. A small fraction of luminal cancers are positive for HER2 and called luminal HER2. PR expression usually follows the ER expression but can be low in some cases where prognoses tend to be poorer. Two major subtypes, besides luminal cancers, are HER2-enriched and triple-negative (ER-/PR-/HER2-) [2]. HER2-enriched cancers show amplification and high expression of the ERBB2 gene and several other genes of the ERBB2 amplicon. Breast cancer classification based on gene expression profiles has also been proposed [3]. Such molecular classifications of cancers are of great clinical importance, since they may provide molecular bases for predicting the tumors' prognoses and/or responses to therapy [2].

Altered patterns of DNA methylation are found in cancer cells, and their values as potential biomarkers for diagnoses and prognoses have been explored [4]. For example, attempts to correlate genome-wide DNA methylation patterns in breast cancers and their prognoses have been made [5–7]. Since epigenetic silencing of tumor suppressor genes is involved in carcinogenesis, inhibitors of certain epigenetic regulators, such as DNA methyl transferases (DNMTs) and histone deacetylases (HDACs), have been tested in cancer therapy with promising results [8–10]. In fact, two DNMT inhibitors, 5-azacytidine and 5-aza-2′-deoxycytidine (decitabine), have already been approved by FDA for treatment of myelodysplastic syndrome [11, 12].

**Figure 1:**
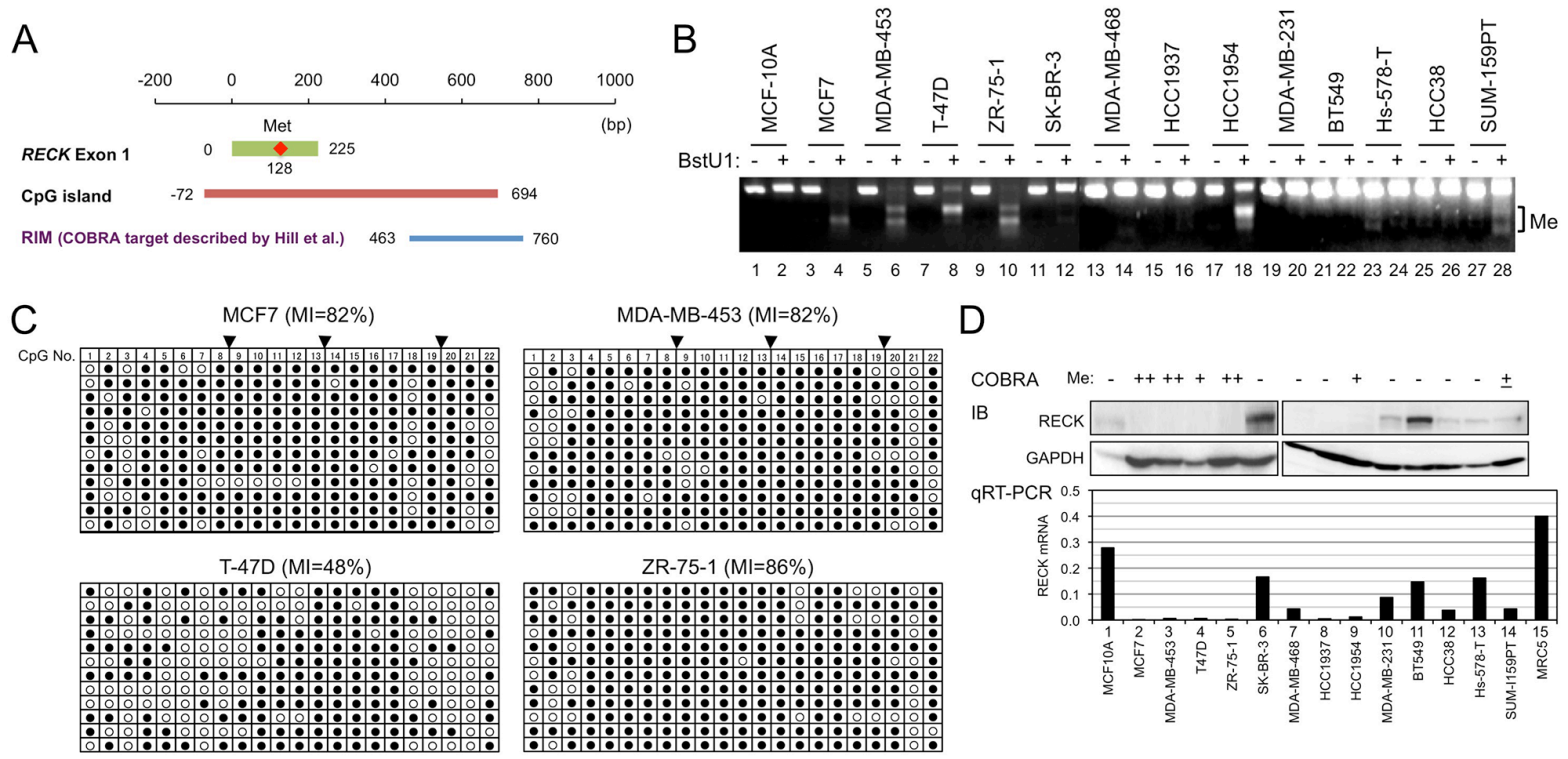
*RECK* methylation and expression in breast cancer cell lines **A.** Positions of exon-1 (green line), first methionine (red diamond), CpG island (orange line), and the COBRA target region described by Hill et al. (RIM, blue line) [5] in which three BstU1 sites (vertical lines) exist. Nucleotide sequence of this region is shown in Supplementary Figure S1. **B.** *RECK* CpG methylation (RIM) in 14 cell lines detected by COBRA. Appearance of smaller bands after BstU1-digestion (even number lanes) indicates methylation of the BstU1 site (CGCG). **C.** RIM determined by clone-sequencing. The PCR products used for COBRA were cloned into bacterial plasmids, and 12 independent plasmid clones (represented by each line) sequenced. Methylation status of 22 CpG di-nucleotides (column 1-22) within the amplified area is shown using open (non-methylated) or filled circles (methylated). Solid triangles on the top indicate BstU1 sites (Supplementary Figure S2, red triangles). **D.** The levels of RECK protein detected by immunoblot assay (IB; upper autoradiographs) and *RECK* mRNA detected by qRT-PCR (lower bar graph). GAPDH was used as an internal control in IB; RNA from normal human fibroblasts (MRC5) was used as a positive control in qRT-PCR. Top line indicates the RIM status estimated from B and C.

*RECK*, initially isolated as a transformation suppressor gene by cDNA-expression cloning, encodes a GPI-anchored glycoprotein with activities to downregulate or inhibit multiple metalloproteinases [13, 14]. Reduced *RECK* expression is found in various types of tumors, and the levels of residual RECK expression in tumors tend to correlate with better prognoses [15, 16]. In gene-focused studies, extensive methylation of *RECK* CpG island has been found in cancers of the colon, stomach, liver, and lung, and its correlation with poorer prognoses detected [17–22]. Importantly, in a non-biased, genome-wide methylation study, Hill and colleagues detected *RECK* as one of several loci whose methylation inversely correlated with relapse-free survival among breast cancer patients [5]. They employed an assay called Combined Bisulfite Restriction Analysis (COBRA) [23] for rapid detection of CpG methylation; in the case of *RECK*, they used a set of PCR primers amplifying an intron-1 region of its CpG island (see Figure 1A, bottom line). We refer to the methylation of this region “RIM” (**RECK** Intron-1 Methylation) in this paper to distinguish it from a newly examined region (see below).

Involvement of DNMT3B in oncogene-induced *RECK* gene silencing [24] and induction of *RECK* expression by DNMT inhibitors [17, 20, 21, 24–26] have been reported. *RECK* activation by an HDAC inhibitor, trichostatin A (TSA), has also been observed in several systems [27–32], suggesting the involvement of histone modifications. Besides these epigenetic regulations, multiple other mechanisms of *RECK* downregulation, such as signaling triggered by growth factors, cell density, oncoproteins, hypoxia, and post-transcriptional inhibition by microRNAs, have also been reported [29, 30, 32–34].

In the present study, we designed a new set of primers amplifying a region of *RECK* CpG island containing the proximal promoter and exon-1 (see Figure 3A, bottom line); we refer to the methylation of this region “RPM” (*RECK* Promoter/exon-1 Methylation). Our data indicate the utility of RIM and RPM as distinctive molecular markers useful for sub-grouping luminal breast cancers and their potential values in predicting the prognoses and drug-sensitivity of breast cancers.

## RESULTS

### *RECK* CpG methylation in breast cancer cell lines

Focusing on the intro-1 region of the *RECK* CpG island, Hill and colleagues found a significant inverse correlation between its methylation (RIM) in tumor tissues and relapse-free survival of the patients [5]. Their data also suggested the prevalence of RIM in the tumors positive for ER (*P*=0.013) and those at relatively early stages (*P*=0.022) (Supplementary Table S2).

To confirm and extend these important findings, we applied the same COBRA protocols to analyze RIM in 16 cell lines, one derived from normal mammary epithelium (MCF-10A) and 15 from breast cancers of various subtypes (Figure 1B; Supplementary Figure S1). RIM was detected in 8 of 15 cancer-derived cell lines (53%) but not in MCF-10A (Table 1). The COBRA results were confirmed by sequencing individual PCR products cloned into a bacterial plasmid (Figure 1C and data not shown). The pattern of RIM distribution among breast cancer subtypes is largely recapitulated in this small set of cell lines: significant enrichment of RIM in luminal cancers (5 of 5) and its paucity in triple negative cancers were detected (1 of 7; Table 1A, 1B, “RIM” column). RECK expression (both mRNA and protein) is low in cell lines positive for RIM (Figure 1D), suggesting the involvement of RIM in *RECK* silencing. These data also suggest the value of these cell lines in studying how RIM affects *RECK* expression and the properties of breast cancer cells.

**Table 1:** Summary of data on breast cancer-derived cell lines

A							
Cell line	Subtype	Medium^1)^	ER	PR	HER2	RPM (%)	RIM (%)
MCF10A	Normal	M	−	−	−	−	−
MCF7	Luminal	D	+	+	−	1.1	82
T-47D	Luminal	R	+	+	−	55	48
ZR-75-1	Luminal	R	+	+/−	−	90	86
KPL-1	Luminal	R	+	−	−	−	+^2)^
KPL-3C	Luminal	R	+	−	−	−	+^2)^
MDA-MB-453	HER2	D	−	−	+	90	82
SK-BR-3	HER2	R	−	−	+	−	−
HCC1954	HER2	R	−	−	+	0.9	74
MDA-MB-468	TN	R	−	−	−	−	−
HCC1937	TN	D (HG)	−	−	−	−	−
MDA-MB-231	TN	D	−	−	−	0.3	−
BT549	TN	R	−	−	−	−	−
HCC38	TN	R	−	−	−	−	−
Hs578-T	TN	DI	−	−	−	−	−
SUM-159PT	TN	F	−	−	−	−	17

1) M, MEGM; D, DME + 10% FBS, R, RPMI + 10% FBS; HG, high glucose; DI, DME + 10% FBS, 10 μg/ml bovine insulin; F, Ham's F12 + 5%FBS, 5 μg/ml insulin,1 μg/ml hydrocortisone,10 mM HEPES

2) Analyzed only by COBRA (See Supplementary Figure S1).

**Table:** 

B						
	Sub-total		RPM		RIM	
	n	Fraction (%)	n	P	n	P
Normal	1	6	0	NS (0.63)	0	NS (0.32)
Luminal	5	31	2	NS (0.22)	5	0.025
HER2	3	19	1	NS (0.52)	2	NS (0.56)
TN	7	44	0	NS (0.20)	1	NS (0.059)
Total	16	100	3		8	

P was assessed by Pearson's chi-squared test. NS, not significant

### Effects of DNMT inhibitor and HDAC inhibitor on *RECK* expression

**Figure 2:**
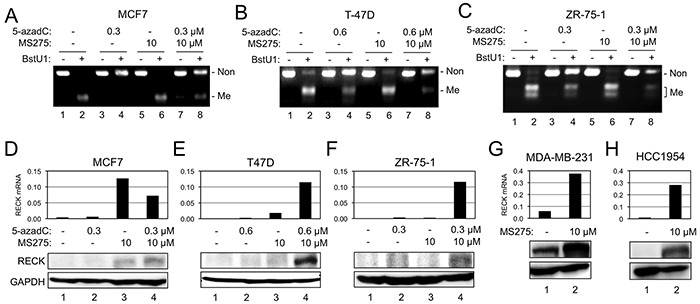
Effects of 5-azadC and MS275 on RIM and RECK expression **A–C.** RIM detected by COBRA in three breast cancer cell lines [MCF7 (A), T-47D (B), or ZR-75-1 (C)] after incubation under indicated conditions. **D–F.** Effects of 5-azadC and/or MS275 on the level of *RECK* mRNA detected by qRT-PCR (upper bar graph) and RECK protein detected by immunoblot assay (lower autoradiographs) in MCF7 (D), T-47D (E), or ZR-75-1 (F). **G, H.** Effects of MS275 on the level of RECK mRNA and protein in two breast cancer cell lines: MDA-MB-231 (G) and HCC1954 (H).

To test the effects of CpG methylation on RECK expression, we treated some of these cell lines with a DNMT inhibitor, 5-aza-2′-deoxycytidine (5-azadC), and determined the levels of *RECK* mRNA and protein. We also examined the effects of an HDAC inhibitor, MS275, which we found to induce RECK expression in some other cell lines, such as those derived from human fibrosarcoma (Yoshida et al., unpublished). First, we confirmed the effects of 5-azadC to reduce RIM in three luminal cell lines (MCF7, T-47D, ZR-75-1) by COBRA; the increased intensity of full-length band and decreased intensity of smaller bands in BstU1-digested samples after 5-azadC-treatment (Figure 2A–2C, compare lane 4 to lane 2) indicate successful DNA demethylation. MS275, on the other hand, exhibited little effects on RIM (Figure 2A–2C, compare lane 6 to lane 2). In these cell lines, 5-azadC alone (namely, demethylation alone) showed little effect on RECK expression (Figure 2D–2F, bar/lane 2). In two RIM-positive cell lines, T-47D and ZR-75-1, combination of two inhibitors (5-azadC and MS275) was effective in activating RECK expression (Figure 2E, 2F, bar/lane 4) while MS275 alone was not so effective (Figure 2E, 2F, bar/lane 3). In a RIM-negative cell line, MDA-MB-231, MS275 alone was sufficient to induce robust RECK expression (Figure 2G), supporting the idea that RIM may be useful in predicting whether or not demethylation is prerequisite for MS275-induced RECK expression. The response of RECK in the RIM-positive MCF7 cells, however, did not support this idea; MS275 could upregulate *RECK* mRNA to some extent but 5-azadC failed to enhance this effect in MCF7 (Figure 2D, bar/lane 3, 4). Survey of additional cell lines yielded another example of RIM-positive cell line, HCC1954, in which RECK could be upregulated by MS275 alone (Figure 2H) although the methylation seems to be incomplete in this case (Figure 1B, lane 18).

### *RECK* promoter/exon-1 methylation (RPM) in breast cancer cell lines

**Figure 3:**
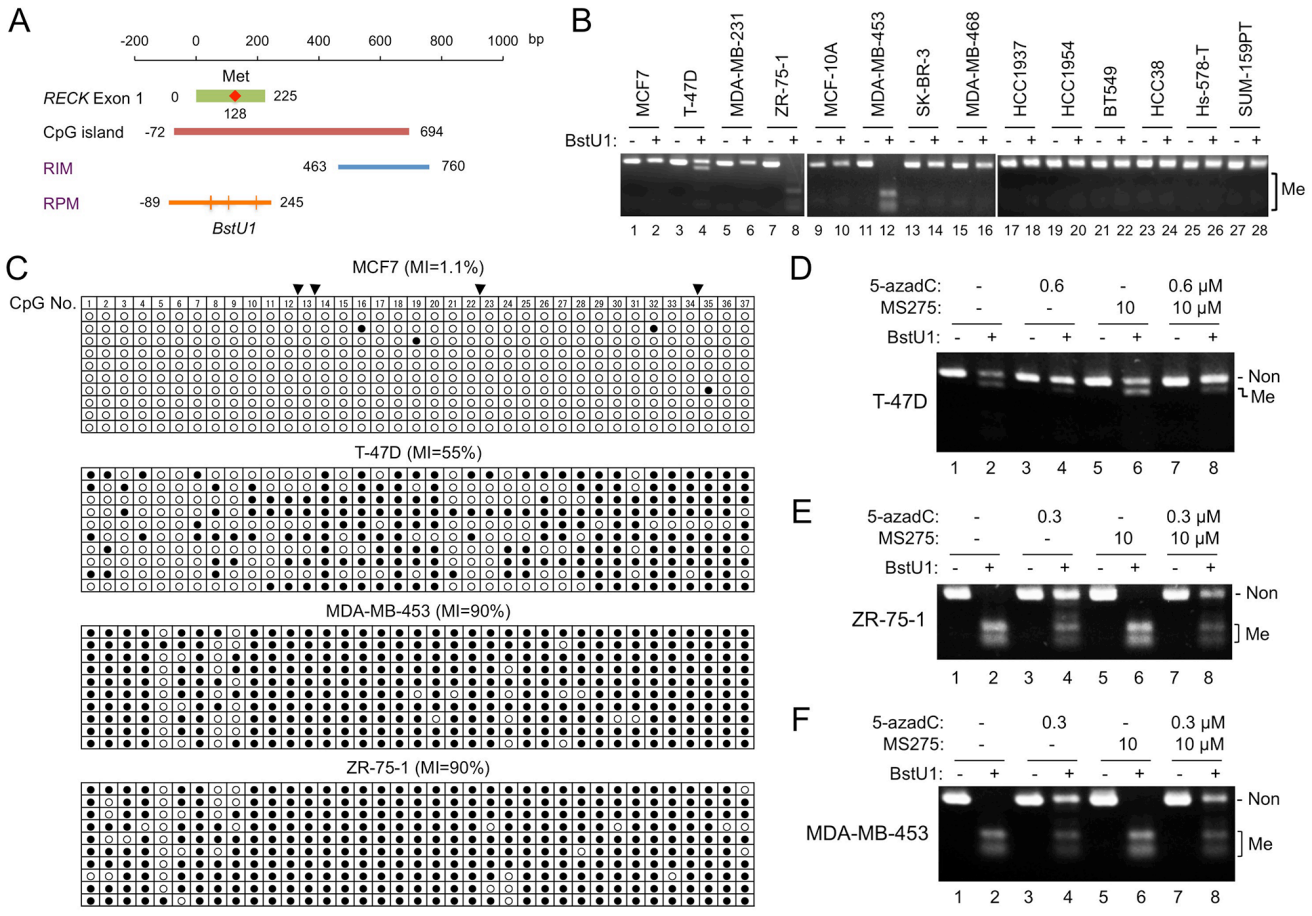
*RECK* gene methylation detected by new COBRA primers **A.** Position of RPM (orange line) with respect to the *RECK* CpG island (brown line) and the original COBRA target (RIM; blue line). Four BstU1 sites (vertical lines), two of which are tandem, exist within the RPM region. Nucleotide sequence of this region is shown in Supplementary Figure S3. **B.** RPM status in 14 cell lines detected by COBRA. **C.** RPM status in four cell lines determined by clone-sequencing. Methylation status of 37 CpG di-nucleotides (column 1-37) within the amplified area is shown. Solid triangles on the top indicate BstU1 sites (Supplementary Figure S3, red triangles). **D–F.** Effects of 5-azadC and/or MS275 on RPM status detected by COBRA in T-47D (D), ZR-75-1 (E), and MDA-MB-453 (F). Note the increased full-length band (representing non-methylated allele) and decreased smaller bands (methylated allele) in BstU1-digested samples (even-numbered lanes) after treatment with 5-azadC (compare lanes 4, 8 to lane 2) but not with MS275 (compare lane 6 to lane 2). Non, band representing to non-methylated DNA; Me, bands representing methylated DNA; MI, ratio of methylated CpG to all CpG.

Why MS275 could upregulate RECK expression in some RIM-positive cells but not others? To test the possibility that we were focusing on an area inappropriate for predicting cell's response to MS275, we designed a new set of COBRA primers to monitor methylation of more upstream region of *RECK* CpG island (RPM; Figure 3A, bottom line). Among the 15 breast cancer cell lines, three (T-47D, ZR-75-1, MDA-MB-453) were positive for RPM (Figure 3B; Table 1); the results were confirmed by clone-sequencing (Figure 3C). As expected, RPM could be reduced by 5-azadC in these cell lines (Figure 3D–3F, compare lane 4 to lane 2) but not by MS275 (Figure 3D–3F, compare lane 6 to lane 2).

Importantly, the extent of RPM at the sequence level (Figure 3C) differs dramatically between MCF7 (1.1%) and two other luminal cell lines, T-47D (55 %) and ZR-75-1 (90%). The other MS275-responsive cell line HCC1954 (Figure 2H) was also RPM-negative (Figure 3B, lane 20). Thus, RPM better predicts the cells' response to MS275 than RIM. In this small set of cell lines, however, no significant association of RPM to certain tumor subtype could be detected (Table 1B, “RPM” column).

### RPM and RIM in breast cancer tissues

**Figure 4:**
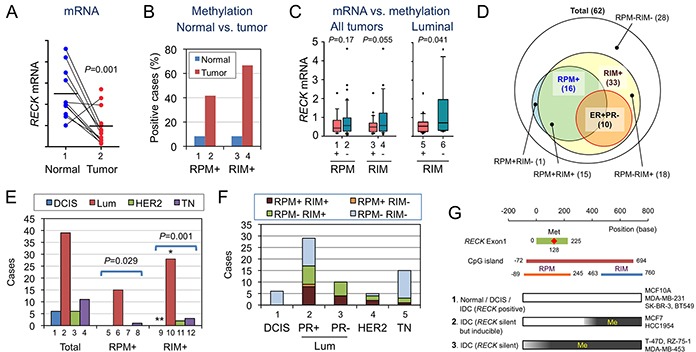
Statuses of *RECK* mRNA, RPM, and RIM in human breast tissues **A.** *RECK* mRNA in normal and tumor tissues from the same patients (n=14). **B.** Proportions of RPM+ and RIM+ cases among the normal (blue) and tumor (red) tissues. **C.** Relationship between the status of RPM or RIM and the level of *RECK* mRNA. **D.** Relationship among breast cancer subgroups exhibiting different RPM/RIM statuses. Their relationship with ER+PR- tumors is also shown. **E.** Distribution of molecular subtypes [DCIS, luminal (Lum), HER2-enriched (HER2), and triple negative (TN)] among total (bars 1-4), RPM-positive (bars 5-8), or RIM-positive (bars 9-12) breast cancer cases. **F.** Distribution of tumors with different RPM/RIM statuses in each molecular subtype. **G.** Three classes of cells with different RPM/RIM statuses. [1] When RPM and RIM are negative, *RECK* is likely to be expressed, albeit at lower levels in tumors. [2] When methylation is confined to RIM, *RECK* is silent but inducible by MS275. [3] When both RPM and RIM are positive, *RECK* is silent and insensitive to MS275 unless the CpG island has been demethylated. Some cell lines of each class are listed on the right-hand side.

To better understand the biological and clinical significance of above findings in cell lines, we compared RPM, RIM, and the level of *RECK* mRNA in tissue samples from 62 breast cancer patients (results summarized in Figure 4 and Table 2; representative data shown in Supplementary Figures S4 and S5). Several findings of potential importance were noted. First, comparison between matched pairs of normal and tumor tissues (n=13) indicated significant downregulation of *RECK* mRNA (*P*=0.001; Figure 4A) and prevalence of *RECK* CpG methylation, both RPM and RIM, in tumor tissues (Figure 4B, bar 2, 4). Second, when 62 tumor samples were divided into two groups based on *RECK* methylation status (RPM or RIM), the average level of *RECK* mRNA was lower in the RIM+ group than the RIM- group, although the difference was not statistically significant (P=0.055; Figure 4C, bar 3, 4). *RECK* mRNA was relatively abundant in luminal tumors (Supplementary Figure S6A), and within this subtype, significant correlation between RIM and lower *RECK* expression was detected (P=0.041; Figure 4C, bar 5). Third, as we found in cell lines (Table 1B, 1A), frequency of RIM among tumor samples (33 of 62 cases; 53%) was higher than that of RPM (16 of 62; 26%), and majority of RPM+ tumors (15 of 16; 94%) were also RIM+ (Figure 4D). Fourth, both RPM and RIM were prevalent in luminal/ER+ tumors (Figure 4E; Table 2). Fifth, *RECK* CpG methylation (both RPM and RIM) was negative, althogh *RECK* mRNA was minimal, in DCIS (n=6; Table 2; Supplementary Figure S6A). Sixth, all the ER+PR- tumors were positive for RIM (10 of 10; Table 2). Seventh, *RECK* CpG methylation, especially RIM, correlates with higher tumor stages and histological grades (Table 2), supporting the idea that the *RECK* silencing (associated with RIM) contributes to the malignant behaviors of breast cancer cells.

**Table 2:** Clinicopathological features and methylation at two regions of *RECK* CpG island (RPM and RIM) in breast cancer tissues

			n	RPM				RIM			
				Number		Ratio	P	Number		Ratio	P
				+	−	RPM+/n (%)		+	−	RIM+/n (%)	
Total			62	16	46	26		33	29	53	
Age	<55		20	4	16	20	NS (0.47)	10	10	50	NS (0.73)
	>55		42	12	30	29		23	19	55	
Subtype	DCIS		6	0	6	0	NS (0.13)	0	6	0	0.00014
	Lum (ER+)	PR+	28	9	19	32		16	12	57	
		PR-	10	4	6	40		10	0	100	
	HER2		5	2	3	40		4	1	80	
	TN		15	1	14	7		3	12	20	
ER	+		40	15	25	38	0.014	29	11	73	0.00087
	−		16	1	15	6		4	12	25	
PR	+		30	10	20	33	NS (0.32)	19	11	63	NS (0.27)
	−		26	6	20	23		14	12	54	
HER2-enrichied	+		5	2	3	40	NS (0.44)	4	1	80	NS (0.19)
	−		51	14	37	27		29	22	57	
Menopause	Pre		17	4	13	24	NS (0.80)	9	8	53	NS (0.98)
	Post		45	12	33	27		24	21	53	
T stage	Tis		7	1	6	14	0.037	1	6	14	0.018
	T1/2		47	10	37	21		25	22	53	
	T3/4		8	5	3	63		7	1	88	
Nodal status	+		28	5	23	18	NS (0.10)	15	13	54	NS (0.48)
	−		27	10	17	37		17	10	63	
Distant Metastasis	+		3	1	2	33	NS (0.61)	3	0	100	NS (0.14)
	−		53	15	38	28		30	23	57	
Grade	DCIS		6	0	6	0	NS (0.15)	0	6	0	0.0055
	1		13	2	11	15		5	8	38	
	2/3		43	14	29	33		28	15	65	

P was assessed by Pearson's chi-squared test. NS, not significant; Tis, tumor in situ

### Effects of MS275 on *RECK* promoter

How does MS275 upregulate *RECK* in RPM-negative breast cancer cells? We confirmed that MS275 increased the level of acetylated histones, as monitored using acetylated H4 (Ac-H4), in three cell lines: MCF7 (RPM-RIM+), T-47D (RPM+RIM+), and MDA-MB-231 (RPM-RIM-) (Supplementary Figure S7B–S7D, lanes 1, 2). As detected by chromatin immunoprecipitation (ChIP) assay, MS275 increased the amount of Ac-H4 bound to three regions (termed P1, P2, and E1; Supplementary Figure S7A) upstream of, or containing, *RECK* exon-1 (Supplementary Figure S7B–S7D, lanes 5, 6). The increase (especially in E1) seems to be modest in T-47D cells (RPM+) (Supplementary Figure S7C, lanes 5, 6). Second, association of HDAC1, a known target of MS275, to the *RECK* promoter was confirmed by ChIP assay (Supplementary Figure S7E, S7F). Relative intensity of 3 ChIP bands differed between cell lines: P1 was most intense in T-47D while E1 was most intense in MDA-MB-231, although biological significance of this difference is unclear. Third, knockdown of HDAC1 using two siRNAs (#1, #2), resulted in RECK-upregulation in MDA-MB-231 cells (Supplementary Figure S7G, lanes 4, 5). These data are consistent with the idea that MS275 upregulates RECK (in RPM-negative breast cancer cell lines) by inhibiting HDAC1, thereby increasing the acetylation of its targets.

### Biological significance of *RECK*-inducibility

**Figure 5:**
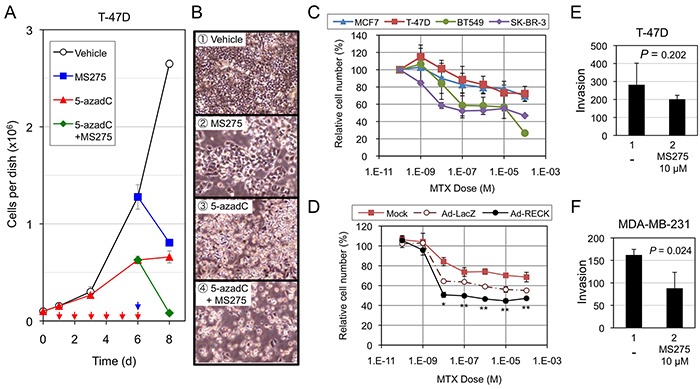
Effects of *RECK*-activation on the growth/survival of breast cancer cell lines **A, B.** Effects of MS275, 5-azadC, or both on the growth/survival of T-47D cells. The cells (1×10^5^/35-mm dish) plated on day 0 were incubated from day 1 to day 6 in growth medium or the medium containing 5-azadC (0.6 μM) with daily medium changes (red arrows), and then exposed to the same medium without or with MS275 (10 μM; blue arrow) from day 6 to day 8. Cell number was estimated by SF assay on day 1, 3, 6, and 8. Bar graph (A) represents mean ± s.e.m. (n=3). Representative micrographs at day 8 are also shown (B). **C.** Effects of MTX on four breast cancer cell lines. The cells were exposed to indicated concentrations of MTX for 48 h, and their number estimated by SF assay. The data represent mean ± s.e.m. (n=3). **D.** Effects of acute *RECK* expression on MTX-sensitivity of T-47D cells. T-47D cells were kept uninfected (Mock) or infected (MOI=600) with either control adenovirus (Ad-LacZ) or *RECK*-expressing virus (Ad-RECK). After 24 h, the cells were replated onto 96-well plates (1 × 10^4^ cell/well), incubated for 16 h, exposed to MTX at various concentrations (0-10^−4^ M; triplicate) for 48 h, and then subjected to SF assay. Data (mean±SD) are presented as their ratios to the vehicle-treated controls. **E, F.** Effects of MS275 on Matrigel invasion of T-47D (E; 20-h incubation) or MDA-MB-231 cells (F; 16-h incubation). The graph represents mean±s.e.m. (n=3).

RPM was inversely correlated with the cells' ability to express *RECK* in response to MS275 (Figure 2; Table 1). Is there any biological significance or clinical relevance in this finding? To address this issue, we first assessed the effects of MS275, 5-azadC, or both on the growth/surival of T-47D cells (RPM+RIM+; Figure 5A, 5B). Although the growth was suppressed to some extent by treatment with MS275 alone or 5-azadC alone (Figure 5A, blue and red lines; Figure 5B-2, 3), almost complete cell killing was observed only after combined treatment (Figure 5A, green line; Figure 5B-4).

Next, we treated four cell lines that exhibit different *RECK* methylation patterns [T-47D (RPM+RIM+), MCF7 (RPM-RIM+), BT549 and SKBR3 (RPM-RIM-)] with a RECK-activating anticancer drug, methotrexate (MTX) [35]. T-47D (RPM+) showed lowest sensitivity to this drug (Figure 5C; red line). Acute expression of RECK using adenoviral vector made T-47D cells susceptible to MTX (Figure 5D, black line), suggesting possible contribution of *RECK* in MTX-induced growth inhibition.

Finally, we subjected two cell lines, T-47D and MDA-MB-231, to Matrigel invasion assay in the absence or presence of MS275 (Figure 5E, 5F) and found significant suppression of invasion by MS275 in MDA-MB-231 cells (RPM-; Figure 5F) but not in T-47D cells (RPM+; Figure 5E). Taken together, these results support the idea that *RECK*-inducibility, as predicted by RPM, may be useful in predicting effectiveness of certain drugs, especially those activating *RECK* expression, in suppressing malignant growth and behaviors of breast cancer cells.

## DISCUSSION

Previous study by Hill et al. [5] revealed a strong correlation between RIM found in tumor tissue and relapse of the disease, which underscored the critical role for *RECK* in tumor suppression. Practical value of RIM as a prognostic marker, however, depends on the availability of therapeutic options for subgroups of the disease to be identified, especially the one with higher risk. RPM developed in this study may be of value in this respect, since it provides information on drug sensitivity. For instance, RIM was positive in all cases (n=10) of ER+PR- tumors examined in this study, while RPM was positive only in 4 cases (Table 2). Similarly, a half of all RIM-positive luminal tumors (n=26) were also RPM-positive. According to our findings *in vitro* (Figure 2), treatment with both 5-azadC and MS275 may be required to upregulate RECK in RPM-positive tumors whereas MS275 alone may be sufficient to induce RECK in RPM-negative tumors. Although the exact biological responses of these particular tumors are unknown, our data *in vitro* (Figure 5) as well as several previous studies indicate its beneficial effects (tumor suppression). Hence, the tumors that require extensive therapies (about a half of RIM-positive cases) may be identified using RPM, in conjunction with RIM and conventional molecular markers (ER, PR, and HER2).

Two lines of new evidence indicate the involvement of CpG methylaiton, especially RIM, in *RECK* silencing. First, RIM was inversely correlated with the level of RECK mRNA/protein among the cell lines (Figure 1; Table 1) and tissue samples (Figure 4A–4C). Second, 5-azadC could convert RIM+ cell lines susceptible to the activity of MS275 to induced *RECK* expression (Figure 2). This mechanism of silencing, however, probably operate only in later stages of mammary carcinogenesis, since RIM was negative in MCF-10A (Figure 1B), most normal tissues (Figure 4B), and DCIS (Table 2) while it was more frequent in high-grade carcinomas (28 of 43; 65%; Table 2).

ER+PR- breast cancers are known to be refractory to anti-estrogen treatment and prone to relapse [36]. A recent study indicate that progesterone inhibits estrogen-induced growth of ER+ breast tumors and that copy number loss of the PR gene is not rare among ER+ breast cancers [37]. The use of DNMT inhibitors in conjunctions with other therapeutic agents, such as anti-estrogens, Cdk inhibitors [38], HDAC inhibitors, or other *RECK*-activating agents [35], may have potential value in treating such tumors.

Our data (Supplementary Figure S7) suggest the involvement of HDAC1 in *RECK* gene repression. Our data also suggest the dominant nature of CpG methylation over this repression (Figure 2). We interpret that although both RPM and RIM are involved in *RECK* gene silencing, RPM probably represent a higher degree of silencing than RIM alone (Figure 4G). The RPM area spans the exon-1 and proximal promoter which harbors numerous potential cis-regulatory elements and has a feature of “CpG island shore” in which the degree of methylation can be influenced by external stimuli, tissue of origin, etc. [4, 39]. This may explain why RPM status varies among RIM-positive tumors and better predicts the cells' ability to express *RECK* in response to MS275. It remains unknown, however, why two groups (RPM+ and RPM-) of RIM-positive luminal cancers arise.

MCF7 and T-47D are commonly used cell lines derived from luminal breast cancer. We found, however, that they differed in the pattern of *RECK* CpG methylation and *RECK* inducibility by chemicals. Molecular mechanisms by which these differences arise are important issues to be addressed in future studies. Some RIM-negative cell lines show different basal levels of RECK expression (Figure 1D). For instance, in MDA-MB-231, basal RECK expression was moderate (Figure 1D, lane/bar 10) and further upregulated by MS275 (Figure 2H), resulting in reduced Matrigel invasion (Figure 5F). Effects of MS275 and other HDAC inhibitors on RECK-expression in, and the behaviors of, other RIM-negative cells may also yield important insights. Studies on the mechanism of action of MS275 in RECK-upregulation may also provide some clues to better understanding how RECK can be repressed in cancer cells.

## MATERIALS AND METHODS

### Cell culture

The mammary epithelial cell line (MCF-10A) and 13 breast cancer cell lines (MCF7, MDA-MB-453, T-47D, ZR-75-1, SK-BR-3, MDA-MB-468, HCC1937, HCC1954, MDA-MB-231, BT549, Hs-578-T, HCC38, SUM-159PT) were obtained from ATCC. Two breast cancer cell lines, KPL-1 and KPL-3C, were described previously [40, 41]. Growth media used for maintainance of these cell lines are shown in Table 1 and were supplemented with fetal bovine serum (FBS; 5% for SUM-159PT and 10% for others).

### Tissue samples

Breast cancer tissues from primary tumor sites were collected through needle biopsy or surgical resection performed in Department of Breast Surgery, Kyoto University Hospital. Prior written informed consents were obtained from all patients. The study protocols were approved by the Ethics Committee for Clinical Research, Kyoto University Hospital (authorization numbers G424). Obtained sample was quickly frozen in liquid nitrogen and stored under −80°C or liquid nitrogen until analyses. Samples collected between May 2011 and June 2014 were used in this study. Pathological characterization was performed as described previously [42]. For molecular analyses, frozen tissues were homogenized using Bead Smash 12 (Waken-yaku, Kyoto, Japan), and the total DNA and RNA was extracted using QIAamp DNA Mini Kit (QIAGEN) and RNeasy Mini Kit (QIAGEN), respectively.

### Methylation analysis of *RECK* gene

COBRA [23] was used to assess the methylation status of *RECK* gene. Bisulfite conversion of genomic DNA was performed using EpiTect Bisulfite kit (Qiagen). Primers for semi-nested PCRs are listed in Supplemental Table S1. Primary PCRs (reaction volume, 25 μl) were performed under the following conditions: 40 cycles of PCR [94°C, 30 s; 50°C, 30 s; 72°C, 60 s] followed by a final extension at 72°C for 5 min. Secondary PCRs were performed using 3 μl of the first PCR product as a template as follows: 45 cycles of PCR [94°C, 30 s; 53°C, 30 s; 72°C, 60 s] followed by a final extension at 72°C, 5 min. PCR products were digested with BstUI (Fermentas, UK) at 37°C for 4 h and resolved by agarose gel electrophoresis (1.5% agarose). For clone-sequencing, PCR products were cloned into pTA2 vector (TOYOBO), and 12 independent clones isolated were sequenced using T3 and T7 primers. Methylation index is defined as the ratio (%) of the number of methylated CpGs to the number all CpGs present in the fragment.

### Quantitative reverse transcription-PCR

Total RNA was extracted using RNeasy Mini Kit (QIAGEN). The levels of specific mRNA were determined using SuperScript III Platinum SYBR Green One-Step qRT-PCR Kit (Invitrogen) with the Mx 3005P RealTime OCR System and Mx Pro software (Stratagene). Primers for *RECK* mRNA and *HPRT* mRNA (internal standard) are listed in Supplemental Table S1. The reaction conditions: an initial reverse transcription [50°C, 5 min; 95°C, 5 min] followed by 45 cycles of PCR [94°C, 15 s; 54°C (*HPRT*) or 64°C (*hRECK*), 40 s; 72°C, 20 s].

### Immunoblot assay

Immunoblot assay was performed as described by Oh et al. using the following antibodies [14]. Primary antibodies: RECK (5B11D12) [13], acetyl-Histone H4 (06-866, Millipore), HDAC1 (5356, CST), and GAPDH (AM4300, Ambion). The secondary antibodies: HRP-conjugated anti-mouse IgG-F(ab')2 monoclonal antibody (A4416, Sigma) and HRP-conjugated anti-Rabbit antibody (7074, CST).

### siRNA transfection

Validated siRNAs for HDAC1 [HSS104725 (#1), HSS104726 (#2)] and a control siRNA (GC Duplex, 462001) were obtained from Invitrogen. The cells (2.5×10^5^/well) were seeded onto six-well plates with DMEM supplemented with 10% FBS without antibiotics. Sixteen hours after plating, siRNA was transfected using Lipofectamine RNAiMAX (Invitrogen) at a final RNA concentration of 100 nM.

### Matrigel invasion assay

FluoroBlok Transwell insert of 8-μm pore size (Corning) were coated by adding 30 ul diluted Matrigel (BD Biosciences; 9.7 mg/ml) and air-dried overnight. The coated inserts were placed on a 24-well plate containing DMEM supplemented with 10% FBS as a chemo-attractant. The cells (2.5×10^4^) were suspended in DMEM containing 0.1% FBS and plated onto the insert. After 16-h incubation, the cells invaded to the lower side of the membrane were stained with Calcein-AM (Dojindo, Japan) for 10 min, and then recorded using Microplate Fluorescence Reader (Molecular Devices, USA).

### Adenovirus-mediated gene transfer

Construction of the control adenoviral vector (Ad-LacZ) and the vector expressing human RECK (Ad-RECK) have been described [43, 44]. Replication-defective viruses were produced from these recombinant cosmids following the protocols of Miyake et al. [45].

### Statistical analyses

Student's t test was used to assess the significance of difference in *RECK* mRNA levels among different groups of tumor samples and in matrix invasion. Relationships between the *RECK* methylation status and clinicopathological parameters were evaluated by Pearson Chi-square tests using JMP software (ver.10, SAS Institute). The difference was considered significant when the *P*-value was smaller than 0.05.

## SUPPLEMENTAry MATERIALS AND METHODS


