# Supplementary material for: Pattern of RECK CpG methylation as a potential marker for predicting breast cancer prognosis and drug-sensitivity
Source: Oncotarget. 2016 Apr 6;7(50):82158–69. doi: 10.18632/oncotarget.8620 (PMC5347682; doi:10.18632/oncotarget.8620)
Supplement: Supplementary file 1 [file oncotarget-07-82158-s001.pdf]

SUPPLEMENTARY MATERIALS AND METHODS

Chromatin immunoprecipitation assay

Chromatin Immunoprecipitation (ChIP) Assay Kit (Upstate Biotechnology, Upstate, NY) was used. In brief, MCF7, T-47D, and MDA-MB-231 cells grown to 80–90% confluence in 6-cm dishes were incubated in the absence or presence of MS275 for 24 h, cross-linked with 1% formaldehyde for 10 min, harvested, and sonicated in SDS-lysis buffer in the presence of protease inhibitor cocktail (Nacalai tesque) to shear DNA to the average size of 500 bp, followed by centrifugation to collect the supernatant (lysate). The lysate was incubated with appropriate antibodies, and the immune complex collected using Protein A agarose (pretreated with salmon sperm DNA to reduce non-specific binding). After washing the beads, the immune complexes were eluted from the beads with 1% SDS, 0.1 M $NaHCO^3$ and heated at 65°C for 4 h to reverse the formaldehyde cross-linking. DNA fragments recovered were purified by phenol-chloroform extraction followed by ethanol precipitation and used for PCR using the primers shown in Supplementary Table S1. PCR products were resolved by agarose gel electrophoresis (1.5% agarose).

SUPPLEMENTARY FIGURES AND TABLES

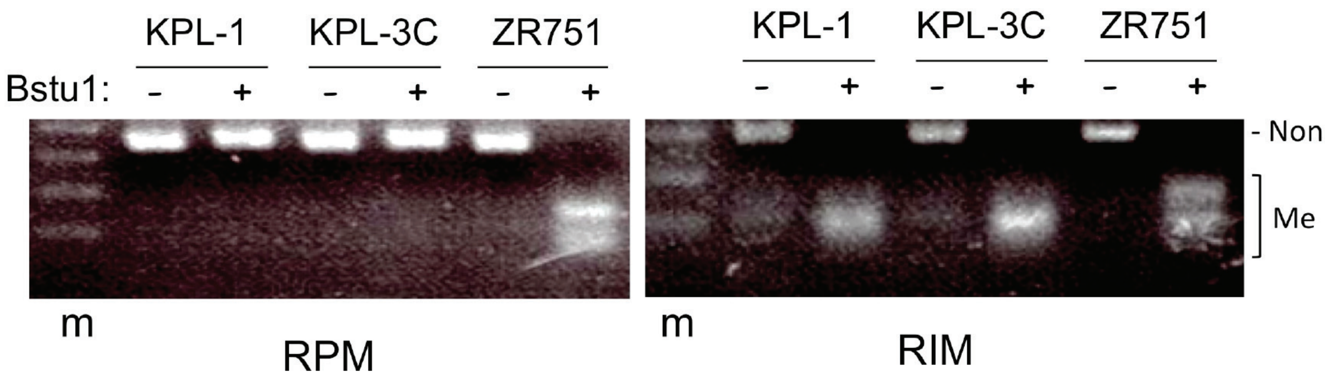

Supplementary Figure S1: *RECK* CpG methylation in three breast cancer cell lines detected by COBRA.

RIM

```
        10         20         30         40         50         60
CACCCACCGA CACGGGCCCC CTCTTCGGCA CTGACCCCTT CGCTTGCCTC TGCTGCTTGT
       (1)        (2)              (3)        (4)
        70         80         90        100        110        120
CAGAAAAGGG TGCGATGCCC CCGCCCAGGA TCGTCGCGAG GTTTAGATGG GATTTCGGAT
                  (5)        (6)       (7)   (8)(9)              (10)
       130        140        150        160        170        180
ACGCAGCCGC CCTACCGCGG CCCTAGTTAG TTATTGTTAC TTGTTACTTG ACCCGCACTT
 (11)       (12)        (13)(14)                                 (15)
       190        200        210        220        230        240
GGTTCATAAC GACCTTGGTG GCGGTGAGCA CTGACGGTCC CCACAGCCCG CGTGGTGGGT
          (16)           (17)          (18)           (19)  (20)
       250        260        270        280        290        300
CCTGGACTCG GGCAGGAGAC ATTAAAAATC AGTAAACCCT GTCCCCCGGG GGCTGGCA
        (21)                                       (22)
```

Supplementary Figure S2: Nucleotide sequence of RIM target region before disulfite treatment. Arrow with blue letters on both ends: positions of primers in the second PCR. Red letters: CpG with its number in parenthesis. Red triangle: BstU1 site.

RPM

```
        10         20         30         40         50         60
TGGGCCATAA CAAAGAGCCC TGGTACGGGG CACGTTCCCG CCCCCGGGAG GTTTTGGAAA
                             (1)     (2)        (3)        (4)
        70         80         90        100        110        120
CACTGTGAGG CAGGGGGCGG GGCTTGAGCG GGCCGCAGCC AGTCACCAAA GGGCCGGGCG
                  (5)         (6)      (7)                  (8)   (9)
       130        140        150        160        170        180
CTGGGGGCGG GGCCTCGCGC GAGCGGCGGC GGTAGCGGCG GCAGCGGCTG CGGCCAAGCT
      (10)     (11)(12)(13) (14)(15) (16)    (17)(18)     (19)      (20)
       190        200        210        220        230        240
GGGTCCGAGC ATCCCGCGGC TCTGGAGCCG CCCGGCCCGG ACATGGCGAC CGTCCGGGCC
    (21)          (22)(23)           (24)   (25)   (26)      (27)  (28)  (29)
       250        260        270        280        290        300
TCTCTGCGAG GTGCGCTGCT CCTTCTGCTG GCCGTGGCGG GGGTCGCGGA GGTGGCAGGG
      (30)      (31)              (32)   (33)   (34)(35)
       310        320        330        340        350        360
GGCCTGGCTC CGGGCAGTGC GGGTGAGTAA CCTCCAGAGC AA
           (36)       (37)
```

Supplementary Figure S3: Nucleotide sequence of RPM target region before disulfite treatment. Arrow with blue letters on both ends: positions of primers in the second PCR. Red letters: CpG with its number in parenthesis. Red triangle: BstU1 site.

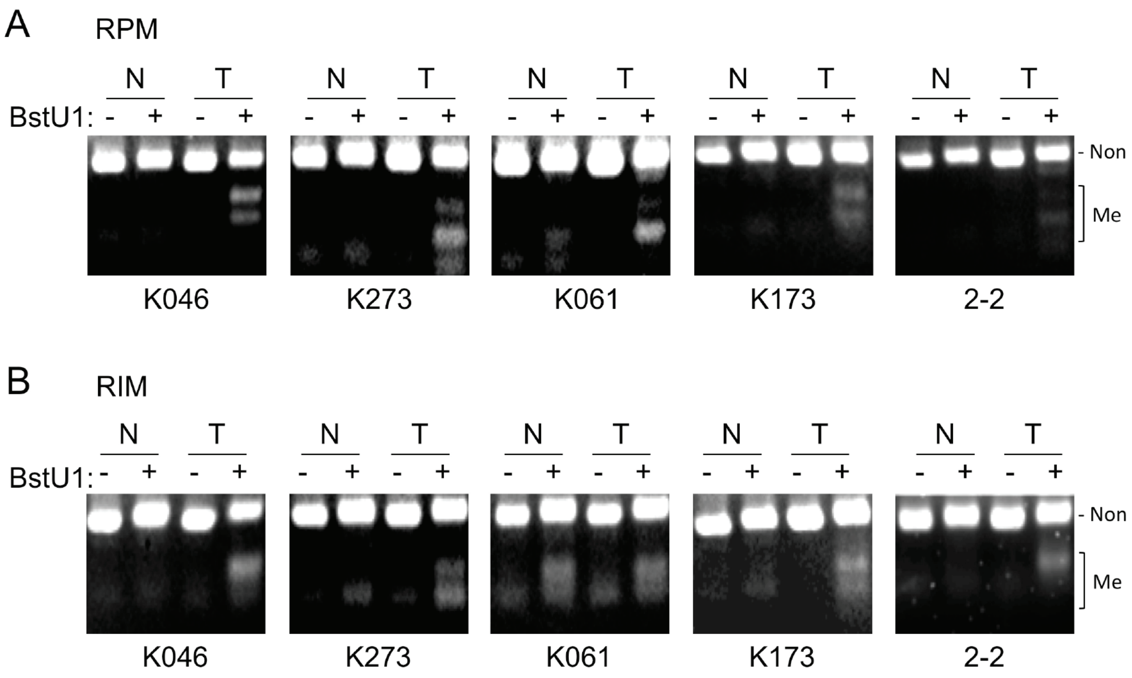

Supplementary Figure S4: ***RECK*** **CpG methylation in 5 breast cancer samples detected by COBRA.**

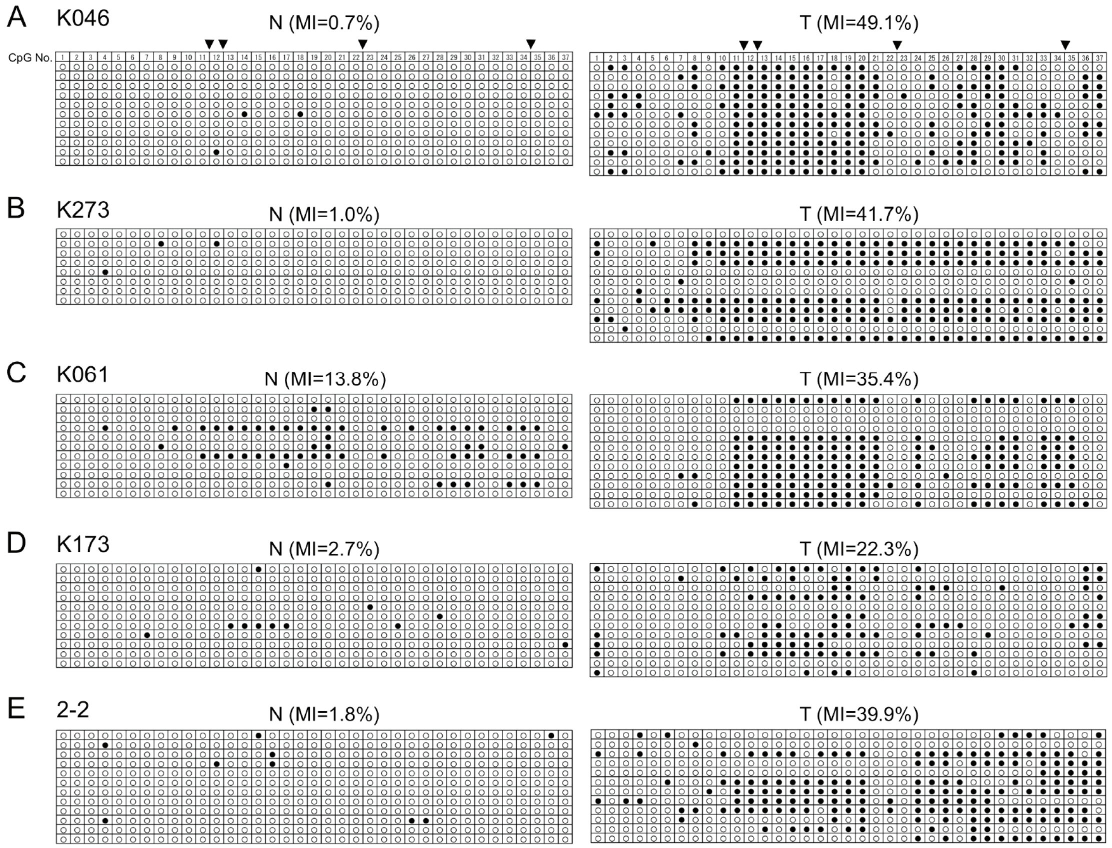

Supplementary Figure S5: ***RECK*** **CpG methylation in 5 breast cancer samples determined by clone-seqencing.**

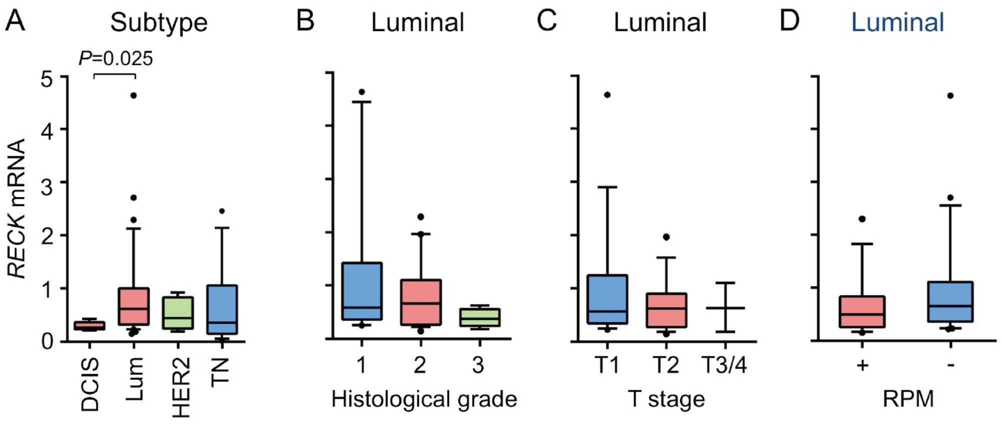

Supplementary Figure S6: *RECK* mRNA in breast tumors of different subtypes (**A**). or in luminal tumors of different histological grades (**B**), T stages (**C**), or RPM statuses (**D**). Among these comparisons, a significant difference was detected only between DCIS and luminal tumor groups in A by Wilcoxon's multiple comparison test.

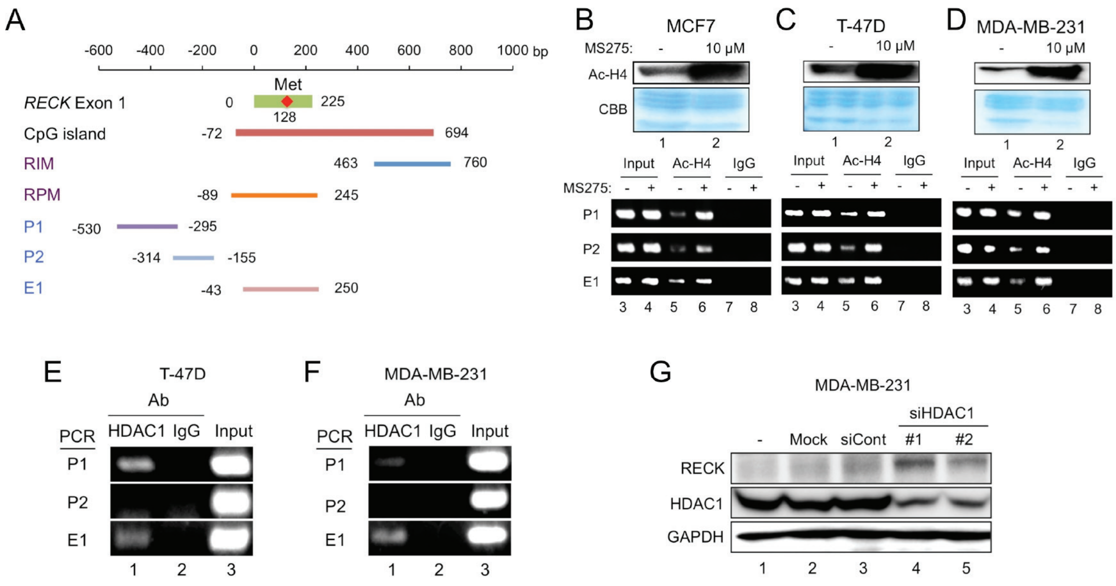

Supplementary Figure S7: Effects of HDAC inhibition. A. Positions of three targets of ChIP-assay used in this study, P1, P2, and E1. **B–D.** Effects MS275 on histone-4 acetylation detected by ChIP-assay in three cell lines: MCF7 (B), T-47D (C), and MDA-MB-231 (D). Immunoblot assay (top panel) together with total protein staining with Coomassie Brilliant Blue (CBB; second panel) indicated increase in the level of total acetylated histone-4 (Ac-H4) after treatment with MS275. ChIP-assay (lower part) indicates the increase in the amount of Ac-H4 bound to P1, P2, or E1 region of *RECK* gene after treatment with MS275 (compare lane 6 to lane 5). **E, F.** Association of HDAC1 detected by ChIP-assay in T-47D (E) and MDA-MB-231 (F). Note the clear signals of HDAC1 associated with the P1 and E1 regions (lane 1). **G.** Positive effects of HDAC1-knockdown by siRNA (lanes 4, 5) on RECK expression (top panel). MDA-MB-231 cells transfected with one of the two HDAC1 siRNAs (#1, #2) were harvested, lysed, and subjected to immunoblot assay with antibodies against RECK (top panel), HDAC1 (middle panel), or GAPDH (bottom panel).

Supplementary Table S1: PCR primers

Assay	Target	Orientation	Sequence
CoBRA	RECK Intron-1	Primary F	ATTTTGTTTAYGTTYGGYGATTTYGGGATT
		Secondary F	TATTTATYGATAYGGGTTTTTTTTYGGTATTGATT
		R	ATCCCRCCCCCRAAAAACAAAATTACTA
	RECK Promoter/ Exon-1	Primary F	ATTTTTTGATTTTATTTTGGGAGAA
		Secondary F	TGGGTTATAATAAAGAGTTTTGGTA
		R	TTACTCTAAAAATTACTCACCC
ChIP	RECK promoter (P1)	F	GACAGAGCGACTCTTGCCTA A
		R	GAACCAAAGGGGCTTCTCTC
	RECK promoter (P2)	F	GAGAGAAGCCCCTTTGGTTC
		R	GAGGATGTCAGAGCTGGGAG
	RECK Exon-1 (E1)	F	TTGGAAACACTGTGAGGCAG
		R	AACCGTTGCTCTGGAGGTTA
qRT-PCR	RECK mRNA	F	GCTGGCAATTTGGTGTGCTCTA
		R	GGGTAAGTGCGCCCATTCTG
	HPRT mRNA	F	CCAGACAAGTTTGTTGTAGG
		R	TCCAAACTCAACTTGAACTC

Supplementary Table S2: Clinicopathological features and *RECK* CpG methylation (Intron-1 region) based on data by Hill et al. (2011)

			n	RIM			P
				Number		Ratio	
				+	-	RIM+/n (%)	
Total			37	10	27	27	
Age	<55		18	4	14	22	NS (0.41)
	>55		19	6	13	32	
Subtype	Lum (ER+)	PR+	16	6	10	38	NS (0.20)
		PR-	2	0	2	0	
	HER2		7	3	4	43	
	TN		12	1	11	8	
ER	+		21	9	12	43	0.013
	-		16	1	15	6	
PR	+		21	8	13	38	NS (0.082)
	-		16	2	14	13	
HER2	+		8	3	5	38	NS (0.45)
	-		29	7	22	24	
Menopause	Pre		23	6	17	26	NS (0.87)
	Post		14	4	10	29	
T stage	<30mm		20	4	16	20	NS (0.24)
	≥30mm		16	6	10	38	
Nodal status	+		29	10	19	34	NS (0.052)
	-		8	0	8	0	
Relapse	+		16	9	7	56	0.00048
	-		21	1	20	5	
Stage	1/2		21	9	12	43	0.022
	3		14	1	13	7	

P was assessed by Pearson's chi-squared test. NS, not significant
